# Co-encapsulation of omega-3 and vitamin D_3_ in beeswax solid lipid nanoparticles to evaluate physicochemical and *in vitro* release properties

**DOI:** 10.3389/fnut.2024.1323067

**Published:** 2024-04-03

**Authors:** Mohammad Shakeri, Runak Ghobadi, Sara Sohrabvandi, Elham Khanniri, Neda Mollakhalili-Meybodi

**Affiliations:** ^1^Department of Food Science and Technology, Faculty of Nutrition Sciences and Food Technology, National Nutrition and Food Technology Research Institute, Shahid Beheshti University of Medical Sciences, Tehran, Iran; ^2^Department of Food Technology Research, Faculty of Nutrition Sciences and Food Technology, National Nutrition and Food Technology Research Institute, Shahid Beheshti University of Medical Sciences, Tehran, Iran; ^3^Department of Food Sciences and Technology, School of Public Health, Shahid Sadoughi University of Medical Sciences, Yazd, Iran

**Keywords:** beeswax, omega-3, simultaneous entrapment, solid lipid nanoparticles, vitamin D_3_

## Abstract

In recent years, lipophilic bioactive compounds have gained much attention due to their wide range of health-benefiting effects. However, their low solubility and susceptibility to harsh conditions such as high temperatures and oxidation stress have limited their potential application for the development of functional foods and nutraceutical products in the food industry. Nanoencapsulation can help to improve the stability of hydrophobic bioactive compounds and protect these sensitive compounds during food processing conditions, thus overcoming the limitation of their pure use in food products. The objective of this work was to co-entrap vitamin D_3_ (VD_3_) and omega 3 (ω3) as hydrophobic bioactive compounds providing significant health benefits in beeswax solid lipid nanoparticles (BW. SLNs) for the first time and to investigate the effect of different concentrations of VD_3_ (5 and 10 mg/mL) and ω_3_ (8 and 10 mg) on encapsulation efficiency (EE). Our findings revealed that the highest EE was obtained for VD_3_ and ω3 at concentrations of 5 mg/mL and 10 mg, respectively. VD_3_/ω3 loaded BW. SLNs (VD_3_/ω3-BW. SLNs) were prepared with zeta potential and size of-32 mV and 63.5 nm, respectively. Results obtained by *in-vitro* release study indicated that VD_3_ release was lower compared to ω3 in the buffer solution. VD_3_ and ω3 incorporated in BW. SLNs demonstrated excellent stability under alkaline and acidic conditions. At highly oxidizing conditions, 96.2 and 90.4% of entrapped VD_3_ and ω3 remained stable in nanoparticles. Moreover, nanoparticles were stable during 1 month of storage, and no aggregation was observed. In conclusion, co-loaded VD_3_ and ω3 in BW. SLNs have the great potential to be used as bioactive compounds in food fortification and production of functional foods.

## 1 Introduction

The incorporation of hydrophobic bioactive components such as essential oils, fat-soluble vitamins, drugs, nutraceuticals, antimicrobials, and flavors into supplements, pharmaceuticals, and functional foods pose many challenges owing to their low solubility in water and inadequate bio-accessibility that need to be mixed with aqueous media to be suitable for oral administration (1, 2). One of the most efficient strategies to overcome the solubility issue and bioavailability is using nano-delivery systems. Since lipid colloidal carriers like waxes or fats have biocompatibility with lipid matrices, they are recognized as suitable nano-delivery systems in the food industry to encapsulate lipophilic bioactive compounds (3). In addition, lipid-based nanocarriers have a high specific surface area, leading to improved bioavailability and biodistribution of loaded bioactives (4, 5).

Nanotechnology has brought out new methods for food processing that aim to enhance physicochemical properties and the stability and availability of nutrients (6). Nanoparticles have remarkable mesoscopic characteristics such as increased surface area, elevated reactivity, minuscule particle size, enhanced strength, quantum effects, and ductility, making them highly sought-after in several industries (7). Flavoring agents, preservatives, encapsulated food components, and other nanoparticles and nanoscale food additives are used to modify the nutritional content and improve the shelf life, scent, and texture of food products (8). At present, encapsulation is viewed as an efficient approach that could increase the intake of sensitive chemicals via products by delaying oxidation and hiding the disagreeable taste of specific components (9).

Lipid-based nanoparticles are known as solid lipid nanoparticles (SLNs), which were first known in 1991 by Müller and gained the attention of researchers worldwide for the delivery of molecules with low bioavailability and poor solubility. SLNs are composed of a solid core containing solid (rather than liquid) lipids with a high melting point dispersed in an aqueous surfactant solution. Lipids used in the manufacture of the nanoparticles are in the solid state at 25°C and the average size of nanoparticles is in the range of 40–1,000 nm (10, 11). SLNs have many advantages over traditional colloidal carriers such as polymeric nanoparticles, emulsions, and liposomes (11, 12). Hence, they have potential applications in the pharmaceutical field and food industry as carriers for antimicrobial compounds or lipophilic bioactives and dermatological and cosmetic preparations (13). Studies have shown that the physicochemical and structural properties of SLNs depend on their ingredients and processes of production. It means that the size of particles, long-term stability, release behavior, loading capacity, and encapsulation efficiency of hydrophobic bioactive components are affected by the SLN formulation in terms of selected surfactants and lipids and their composition (14, 15). Lipid substances used in the fabrication of SLNs should be generally recognized as safe (GRAS). Beeswax is one of the components that can be used as a solid lipid with a melting temperature of 61–67°C for the preparation of SLNs owing to its low toxicity, biodegradability, and low cost (16, 17). In fact, liquids secreted by special wax glands in the abdomen of worker bees are called beeswax, which changes to a solid state in contact with air (18). It consists of hydrocarbons, free fatty acids, saturated and unsaturated linear and complex monoesters, alcohols, and other minor compounds (19). Therefore, due to the hydrophobic properties of beeswax, it is a proper matrix for lipid-soluble compounds.

Co-encapsulation of more than one bioactive component is a suitable technique to increase their functionality and bioactivity compared to a single bioactive by inducing synergistic effects between ingredients. In recent years, co-delivery systems have been widely applied in the food and pharmaceutical industries to maintain the stability of certain active components or cure specific diseases (20, 21). For example, omega-3 fish oil has been shown to have strong oxidative stability when co-encapsulated with α-tocopherol (22). In a study conducted by Xiao et al. (23), it was stated that co-encapsulation of fish oil with garlic essential oil exhibited the highest oxidative stability during 30 days of storage. Similarly, Tchuenbou-Magaia et al. (24) reported that co-encapsulated rutin enhanced the activity and stability of vitamin D_3_ and the chitosan-zein microparticles could be a suitable delivery system for the enrichment of food products with vitamin D_3_.

Omega-3 fatty acids are essential bioactive compounds with physiological functions such as combating neural disorders, preventing cardiovascular diseases, and improving memory processing (25). One of the issues of producing fortified foods with omega-3 fatty acids is the autoxidation of long-chain polyunsaturated fatty acids, which causes rancidity and reduced shelf life (26). Cholecalciferol (vitamin D_3_) is a liposoluble pro-hormone that is involved in the maintenance of calcium and phosphorus homeostasis (27, 28). People with metabolic (hyperparathyroidism and obesity) or gastrointestinal diseases, as well as those who do not receive enough sunshine exposure, often have vitamin D_3_ deficiencies (29). Deficiency of vitamin D_3_ can lead to rickets and osteoporosis. Also, it has an important role in the prevention of diseases such as cancer (30). Since vitamin D_3_ and omega-3 fatty acids (ω3) are hydrophobic compounds, they cannot be easily dispersed into formulations of aqueous food. Therefore, it is necessary to create efficient delivery mechanisms to overcome these obstacles and to enable the incorporation of these bioactive lipids into different functional foods. On the other hand, the co-entrapment of omega-3 and vitamin D_3_ in SLNs has not been evaluated yet. Thus, the present work aimed at the co-encapsulation of fat-soluble vitamin D_3_ (VD_3_) and omega 3 (ω3) in beeswax solid lipid nanoparticles (BW. SLNs) to increase the stability of both these bioactive compounds. The obtained results concerning size, zeta potential, morphology analysis of nanoparticles, encapsulation efficiency, the *in-vitro* release of VD_3_ and ω_3,_ and their stability under different conditions are presented and discussed.

## 2 Materials and methods

### 2.1 Materials

Beeswax (BW) with a melting point of 60–66°C and purity of 99%, vitamin D_3_ (VD_3_), omega 3 (Docosahexaenoic acid), and egg yolk lecithin were purchased from Sigma Chemical Co (St Louis, Mo., United States). Tween-80 was obtained from Merck (Germany). All the solvents used in this research were of the highest commercially accessible grade.

### 2.2 Preparation of VD_3_/ω3-BW. SLNs, VD_3_-BW. SLNs, ω3-BW. SLNs and unloaded BW. SLNs

**Figure 1:**
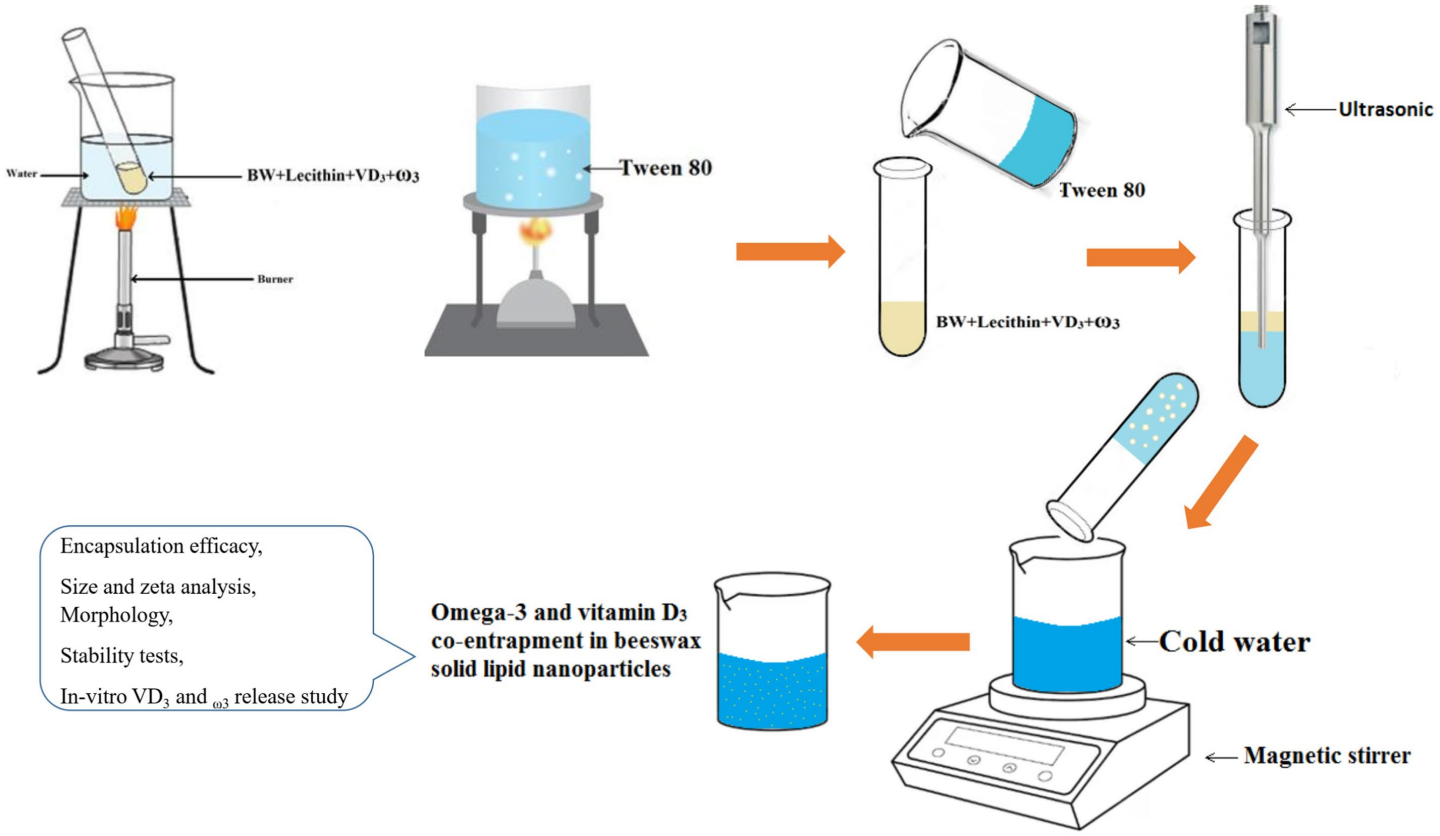
Preparation of beeswax solid lipid nanoparticles.

The production of VD_3_/ω3-BW. SLNs were accomplished using a Nano-emulsion method (31), as shown in Figure 1. Briefly, BW (25 mg), lecithin (7.3 mg), VD_3_ (5 and 10 mg/mL ethanol), and ω3 (8 and 10 mg) were heated to a temperature of 90 ° C in a water bath inside a glass tube with a screw cap. In a similar way, 5 mL of distilled water (aqueous phase) containing (0.8% v/v) tween 80 was heated at 90°C for 10 min and then added to the mixture of molten beeswax and lecithin, which resulted in the formation of an emulsion. The emulsion was gently stirred for 1 min, and an ultrasonic homogenizer was used to disperse it at a frequency of 24 kHz within 2 min. Finally, to facilitate the production of VD_3_/ω3-BW. SLNs, this emulsion was slowly poured into 50 mL of cold water at a temperature of 4°C with a volume ratio of 1:10 using a syringe and stirred with a magnetic stirrer at a speed of 1,100 rpm. Also, this approach was applied to prepare VD_3_-BW. SLNs, and ω3-BW. SLNs with an optimal concentration of VD_3_ and ω3, respectively, and unloaded BW. SLNs (U-BW. SLNs). Table 1 displays the various concentrations of VD_3_ and ω3 used in the production process of VD_3_/ω3-BW. SLNs to determine the best formula, which was used for size and zeta analysis, morphology, and stability tests.

**Table 1:** The different concentrations of vitamin D_3_ and omega 3 utilized in the production of VD_3_/ω3-BW. SLNs to determine the optimal formulation.

Formulation^*^	Vitamin D3 (mg/ml)	Omega 3 (mg)	Size (nm)	Vitamin D_3_ EE (%)	Omega 3 EE (%)
VD_3_/ω3-BW. SLNs	5	8	72.30 ± 2.40^a^	89.70 ± 1.50^a^	81.70 ± 1.90^b^
VD_3_/ω3-BW. SLNs	5	10	63.50 ± 4.00^b^	92.30 ± 1.20^a^	86.30 ± 1.40^a^
VD_3_/ω3-BW. SLNs	10	8	67.80 ± 1.80^ab^	91.80 ± 1.80^a^	82.30 ± 1.50^ab^
VD_3_/ω3-BW. SLNs	10	10	65.10 ± 2.50^ab^	92.70 ± 2.00^a^	85.40 ± 1.80^ab^

*VD_3_-BW. SLNs: vitamin D_3_/omega 3-beeswax solid lipid nanoparticles. Means shown with different small letters (a, b) represent significant differences (*p* < 0.05) in the same columns.

### 2.3 Characterization of unloaded and loaded BW. SLNs

#### 2.3.1 Determining the yield of BW. SLNs

The yield of prepared BW. SLNs was measured by freeze-drying 10 mL of the prepared BW. SLNs. Afterward, the produced nanoparticles were weighed, and the total mass of the sample was determined.

#### 2.3.2 Morphology analysis

The morphology of nanoparticles was evaluated using a scanning electron microscope (SEM) (JEOL LSM5600LV). The centrifugation process was used to purify samples of both unloaded and loaded BW. SLNs. The produced nanoparticles were frozen at −20°C and then dried in a freeze drier. These samples were utilized for analysis by SEM.

#### 2.3.3 Particle size and zeta potential analysis of loaded and unloaded BW. SLNs

The Zetasizer Nano DLS (Silas, France) was used to determine the size and dimension of the samples. The nanoparticles’ zeta potential was measured using a Beckman Coulter Delsa nano zeta potential analyzer. The zeta potential of distributed BW. SLNs in deionized water was analyzed at room temperature. Analyses were performed using aqueous dispersions of unloaded and loaded nanoparticles in triplicate.

### 2.4 Encapsulation efficacy of VD_3_ and ω3

To determine EE%, 1 mL of each nanoparticle solution (VD_3_-BW. SLNs and ω3-BW. SLNs) was centrifuged at 4500 g for 10 min. The upper phase (nanoparticle phase) was extracted and added to 5 mL of acetonitrile. After 30 min of stirring at 1000 rpm, the solution was centrifuged for 10 min at 4500 g. The EE% of VD_3_ and ω3 in VD_3_/ω3-BW. SLNs was determined separately according to the mentioned method. Afterward, absorption at 265 and 220 nm was measured to calculate VD_3_ and ω3 by UV spectrophotometry, respectively. EE% was calculated using the standard curve and the following equation (Eq. 1) (31, 32):


(1) $$EE\%=\left[\begin{array}{l} \mathrm{Total amount of loaded vitamin}\;D_{3}\;\mathrm{or omega}\;3/\\ \mathrm{Initial amount of added vitamin}\;D_{3}\;\mathrm{or omega}\;3 \end{array}\right]\times 100$$

### 2.5 *In-vitro* VD_3_ and ω3 release study

To investigate the *in-vitro* release profile of VD_3_ and ω3 from BW. SLNs through the use of the dialysis method, the VD_3_/ω3-BW. SLNs suspension was put inside the dialysis membranes, which had a molecular weight cut-off of 12,000 Da. Afterward, the dialysis tube was inserted into the phosphate buffer saline (PBS, pH: 7.4) and maintained at 30°C at 550 rpm. At predetermined time intervals, 1 mL of samples were collected from the release medium and the quantity of released VD_3_ and ω3 was evaluated using UV spectrophotometry, as described above to determine the entrapment efficiency. Cumulative release of VD_3_ and ω3 was calculated using the following formula (Eq. 2):


(2) $$\mathrm{Cumulative release}\;\left(\%\right)=\left[\begin{array}{l} \mathrm{Released amount of vitamin}\;D_{3}\;\mathrm{or omega}\;3/\\ \left(\mathrm{Total amount of loaded vitamin}\;D_{3}\;\mathrm{or omega}\;3\right) \end{array}\right]\times 100$$

### 2.6 Stability of loaded VD_3_ and ω3 in BW. SLNs under alkaline and acidic pH

Stability of free vitamin D_3_ (F-VD3), free omega 3 (F-ω3), and VD_3_/ω3 loaded in BW. SLNs were determined in pH values of 2 and 9, which the HCl and PBS were used to adjust the pH of formulations. Samples were maintained in the dark for 7 days at room temperature. Then, 1 mL of each sample was taken, and VD_3_ and ω3 were extracted and quantified by HPLC. The following equation (Eq. 3) was used to determine the stability under different pH values (33):


(3) $$\mathrm{Stability}\;\left(\%\right)=\left[\begin{array}{l} \mathrm{Residual amount of vitamin}\;D_{3}\;\mathrm{or omega}\;3/\\ \mathrm{Original amount of vitamin}\;D_{3}\;\mathrm{or omega}\;3 \end{array}\right]\times 100$$

### 2.7 Stability of loaded VD_3_ and ω3 in BW. SLNs under oxidative condition

Hydrogen peroxide (H_2_O_2_) solution was used to examine the oxidative stability of F-VD_3_, F-ω3, and VD_3_/ω3 loaded in BW. SLNs. Samples of F-VD3, F-ω3, and VD3/ω3-BW. SLNs were prepared with 0.1, 0.5, and 1% of H_2_O_2_. The samples were kept in the dark for 2 h at room temperature. 1 mL of samples were collected, and VD_3_ and ω3 were extracted. Then, their oxidative stability was determined by HPLC and assessed by the following equation (Eq. 4) (33):


(4) $$\mathrm{Oxidative stability}\;\left(\%\right)=\left[\begin{array}{l} \mathrm{Residual amount of vitamin}\;D_{3}\;\mathrm{or omega}\;3/\\ \mathrm{Original amount of vitamin}\;D_{3}\;\mathrm{or omega}\;3 \end{array}\right]\times 100$$

### 2.8 Stability of zeta potential and size of VD_3_/ω3-BW. SLNs after 30 days

BW. SLNs loaded with VD_3_ and ω3 in optimal concentrations were stored in a sealed chamber at 4°C for 30 days to evaluate their storage stability. The zeta potential and particle size of VD_3_/ω3-BW. SLNs were measured using a dynamic light-scattering instrument. Analyses were carried out in triplicate.

### 2.9 Statistical analysis

Data was expressed as mean ± standard deviation of three replicates (*n* = 3). The experimental results were analyzed with SPSS version 24 and subjected to one-way analysis of variance (ANOVA). Duncan’s test was used to calculate the significant differences between mean amounts. In all analyses, *p* < 0.05 was considered as significant.

## 3 Result and discussion

### 3.1 Optimal formulation

The effect of different concentrations of VD_3_ (5 and 10 mg) and ω3 (8 and 10 mg) on entrapment efficiency (EE%) was studied to prepare BW. SLNs with simultaneous high efficiency for loading of VD_3_ and ω3. According to the obtained results in Table 1, the maximum EE of VD_3_ (92.3%) and ω3 (86.3%) was achieved when VD_3_ and ω3 were entrapped in the BW. SLNs simultaneously at concentrations of 5 and 10 mg, respectively. Hence, it was selected as an optimum formulation to perform further tests.

### 3.2 Size, zeta potential, and morphology analysis of nanoparticles

#### 3.2.1 VD_3_/ω3-BW. SLNs

**Figure 2:**
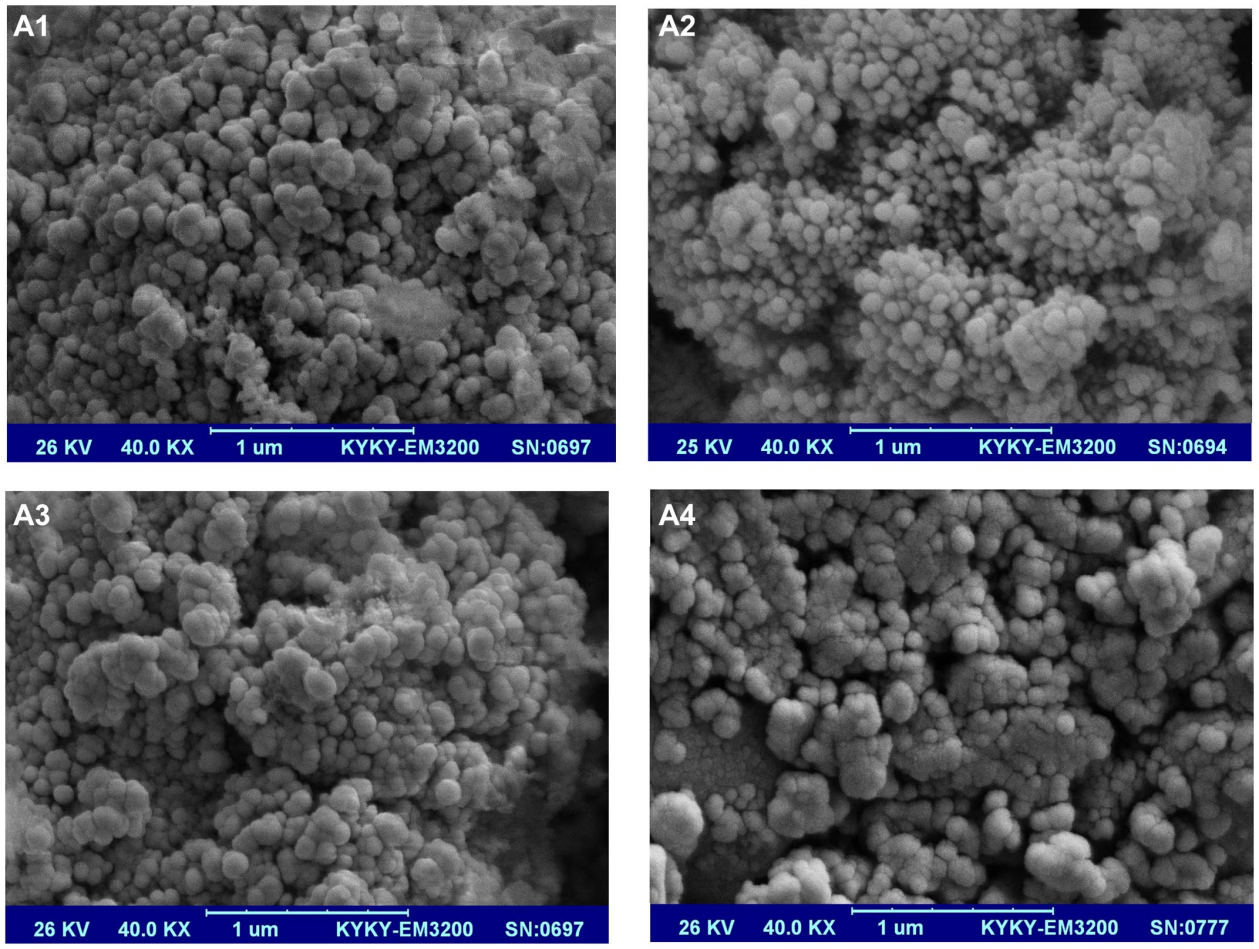
SEM image of VD_3_/ω3-BW. SLNs **(A1)**, VD_3_-BW. SLNs **(A2)**, ω3-BW. SLNs **(A3)** and U-BW. SLNs **(A4)**.

The morphology of nanoparticles was investigated by SEM, as shown in Figure 2. As can be seen, the prepared optimum nanoparticles had a spherical form, and their size was 63.5 ± 4 nm. Nanoparticles’ zeta potential was-32 ± 1.6 mV (Table 2). No aggregation or agglomeration of nanoparticles was seen in the stable aqueous solution of optimal formulation of VD_3_/ω3-BW. SLNs. It seems that beeswax nanoparticles are a suitable carrier for VD_3_ and ω3. Similarly, Dantas et al. (17) reported that beeswax nanoparticles are capable of high drug loading and have not undergone polymorphic modifications. Also, Mehmood and Ahmed (34) declared that mixed surfactant (Tween 80 and soya lecithin) based nanoemulsions are an effective delivery system for incorporation of vitamin D into food and beverages to overcome the worldwide deficiency of vitamin D.

**Table 2:** Characteristics of unloaded and loaded BW-SLNs with vitamin D_3_ and/or omega 3.

Formulation*	Size (nm)	Zeta potential (mV)	Dispersity	Yield (%)	Vitamin D3 EE (%)	Omega 3 EE (%)
U-BW. SLNs	74.20 ± 3.20^ab^	−32.70 ± 1.10^b^	Monomodal	95.80 ± 1.50^a^	–	–
VD_3_-BW. SLNs	66.20 ± 7.30^bc^	−36.00 ± 1.50^a^	Monomodal	93.10 ± 1.20^b^	67.50 ± 0.70^b^	–
ω3-BW. SLNs	78.60 ± 5.20^a^	−24.00 ± 0.40^c^	Monomodal	94.30 ± 1.40^ab^	–	88.60 ± 1.70^a^
VD_3_/ω3-BW. SLNs (optimized)	63.50 ± 4.00^c^	−32.00 ± 1.60^b^	Monomodal	95.10 ± 0.80^ab^	92.30 ± 1.20^a^	86.30 ± 1.50^a^

* U-BW. SLNs, unloaded beeswax solid lipid nanoparticles; VD_3_-BW. SLNs, vitamin D_3_-beeswax solid lipid nanoparticles; ω3-BW. SLNs, omega 3-beeswax solid lipid nanoparticles; VD_3_/ω3-BW. SLNs, vitamin D_3_/omega 3-beeswax solid lipid nanoparticles. Means shown with different small letters (a, b) represent significant differences (*p* < 0.05) in the same columns.

#### 3.2.2 VD_3_-BW. SLNs

After removing ω3 from the optimal formulation of VD_3_/ω3-BW. SLNs, spherical VD_3_-BW. SLNs were created and analyzed, which their characteristics were summarized in Table 2. The size and zeta potential of nanoparticles were raised to 66.2 ± 7.3 nm and-36 ± 1.5 mV, respectively. Similar trends were observed from a study conducted by Fan et al. (35). It was stated that unloaded salidroside nanoliposomes with a particle size of under 100 nm have no electric charge on their surface, and the loading of salidroside resulted in a significant increase in the zeta potential of nano-liposomes within the range of-10 to-20 mV. They stated that the dipole tropism is produced and, as a result, the surface electric charge of nanoparticles is enhanced by the interaction between choline and the hydroxyl group of salidroside (35). In the present study, it seems that the polar region of phosphatidylcholine could interact with the hydroxyl group of VD_3_ and consequently enhance the zeta potential of VD_3_-BW. SLNs. Nanoparticle suspension in aqueous solution was extremely stable, and no aggregation or agglomeration of nanoparticles was seen (Figure 2A2).

#### 3.2.3 ω3-BW. SLNs

Spherical ω3-BW. SLNs were made, and their size was 78.6 ± 5.2 nm. Nanoparticles were mono-modal, although zeta potential was significantly different between ω3-BW. SLNs and VD_3_-BW. SLNs or VD_3_/ω3-BW. SLNs. Zeta potential of ω3-BW. SLNs was reduced to-24 mV after encapsulation of ω3 into BW. SLNs (Table 2).

#### 3.2.4 U-BW. SLNs

U-BW. SLNs were synthesized with a mono-modal distribution and a diameter of 74.2 ± 3.2 nm in an aqueous solution (Figure 2A4). U-BW. SLNs exhibited a negative surface charge (−32.7 mV) in the zeta potential study, and nanoparticles were stable in suspension. Zeta potential of U-BW. SLNs changed after VD_3_ and ω3 loading. When the VD_3_ was loaded into the BW. SLNs, the nanoparticles’ zeta potential increased from−32.70 ± 1.1 mV (unloaded nanoparticles) to-36 ± 1.5 mV (VD_3_-BW. SLNs). On the other hand, the entrapment of ω3 into the BW. SLNs reduced the zeta potential of unloaded nanoparticles from-32.70 ± 1.1 to-24 ± 0.4 mV in ω3-BW. SLNs. Intriguingly, the nanoparticles’ zeta potential was increased in VD_3_/ω3-BW. SLNs from-24 ± 0.4 to-32 ± 1.6 mV when ω3 and VD_3_ were both simultaneously trapped in the SLNs. The formulations containing only ω3 demonstrated bigger nanoparticles. The size of nanoparticles was reduced by adding VD_3_ to the formulations. Additionally, VD_3_ increased the zeta potential of ω3-BW. SLNs from-24 to-32 mV in VD_3_/ω3-BW. SLNs. The ability of VD_3_ to increase the surface charge of BW. SLNs may be the reason for reducing the nanoparticle size. In accordance with our findings, Xiang et al. (36) mentioned that when the zeta potential of OVA-PEC-VD_3_ (ovalbumin-high methoxyl pectin-VD_3_) nanocomplexes decreased due to the reduction of electrostatic repulsions between the particles, their size increased. In a survey conducted by (37), chitosan nanoparticles loaded with *Salvia officinalis* extract exhibit a lower surface charge (+21.8 to +28.8 mV) than unloaded nanoparticles (+32 mV) that is in agreement with our results when ω3 trapped into BW. SLNs. In our study, the BW. SLNs were more stable in aqueous solution when they had a higher negative surface charge. In accordance with our findings, previous research reported that emulsions with high zeta-potential (positive or negative) indicated more repulsion between particles and were electrically stabilized, whereas coagulation or flocculation could occur between particles with low zeta-potential (38, 39).

### 3.3 Determination of yield and EE

The yield of prepared loaded and unloaded nanoparticles was between 93 and 96%. EE of ω3 in ω3-BW. SLNs and VD_3_/ω3-BW. SLNs was 88.6 ± 5.2 and 86.3 ± 1.5%, respectively (Table 2). Also, EE of VD_3_ in VD_3_-BW. SLNs and VD_3_/ω3-BW. SLNs was 67.5 ± 1.6 and 92.3 ± 1.2%, respectively. However, the EE of VD_3_ in VD_3_/ω3-BW. SLNs was much higher than VD_3_-BW. SLNs. It’s interesting to note that when VD_3_ and ω_3_ were loaded simultaneously into the nanoparticles, the maximum EE for VD_3_ (92.3%) was achieved. According to the findings, the EE of VD_3_ increased whenever ω3 was present in the formulations. The encapsulation effectiveness of pharmaceuticals may increase or decrease depending on factors such as crystallinity and polymorphism of solid lipid-based nanoparticles (40, 41). When ω3 is added to formulations of beeswax-based nanoparticles, it may decrease the melting point of SLNs (42) or alter the crystallinity of solid lipid nanoparticles (43). This may increase the solubility of VD_3_, which could increase its interaction with the beeswax-lecithin lipid phase and, subsequently, the efficacy of entrapment in BW. SLNs. Ahmad et al. (44) fabricated micro and nanoparticles of chestnut starch for co-encapsulation of vitamins D, E, B_1,_ and B_2_. They stated that the highest encapsulation efficiency of vitamin D (46.27%) was obtained when it was encapsulated in nano-sized starch (44). This is attributed to the larger surface area of nanocarriers compared to micron-sized carriers (45).

### 3.4 *In-vitro* release study of VD_3_ and ω3 from BW. SLNs

**Figure 3:**
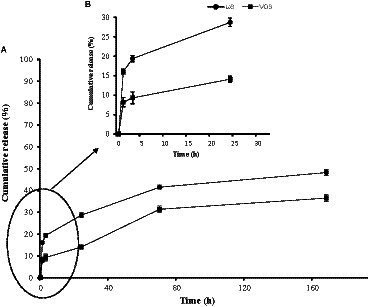
Release profiles of ω3 (-●-) and VD3 (-■-) from BW. SLNs in PBS (pH 7.4) and 550 rpm at 30°C. The first initial burst release (IBR) from 1 to 3 h, second release phase from 3 to 24 h (B) and final third release phase during 24-168 h (A) were observed.

The cumulative release of VD_3_ and ω3 was assessed using the dialysis technique in PBS (pH 7.4), 550 rpm at 30°C (Figures 3A,B). Three release phases were observed and monitored: the first initial burst release phase (IBR), which lasted between 1 and 3 h; the second release phase, which lasted between 3 and 24 h; and the third release phase, which lasted between 24 and 168 h. During 3 h incubation, the IBR of ω3 and VD_3_ was measured. The IBR was 19.4 and 9.3% for ω3 and VD_3_, respectively (Figure 3B). The IBR may be associated with molecules of ω3 and VD_3_ that are poorly adsorbed on the surface of nanoparticles (46). The cumulative release of ω3 and VD_3_ increased to 28.7 and 14.1%, respectively, during the second phase (3–24 h). These data from the second phase demonstrated that ω3 was released into the buffer twice as much as VD_3_. After 168 h, the release of ω3 and VD_3_ reached 48.2 and 36.5% of the total entrapped ω3 and VD_3_, respectively (Figure 3A). It indicates that more than 52 and 63% of ω3 and VD_3_, respectively, remained caught in the BW. SLNs after 168 h. similarly, Hosseini et al. (47) noticed the same early and then gradual emissions of oregano essential oil. Moreover, Shakeri et al. (31) used beeswax nanoparticles to co-encapsulate astaxanthin and carvacrol. They reported that BW. SLNs could slowly release astaxanthin and carvacrol in a buffer, which is in consistent with the present study results. The release of carvacrol and astaxanthin after 168 h was 45.8 and 33.62% of total entrapped carvacrol and astaxanthin (31). Diffusion and hydrolytic degradation (erosion) are two important mechanisms for drug release from SLNs. SLNs degrade in the presence of water by an ester hydrolysis reaction that is acid-catalyzed and reversible (48). A better release profile and higher retention of encapsulated bioactive compounds occur due to the protective effects of encapsulation (49).

### 3.5 Stability of VD_3_/ω3-BW. SLNs under acidic and alkaline pH

**Figure 4:**
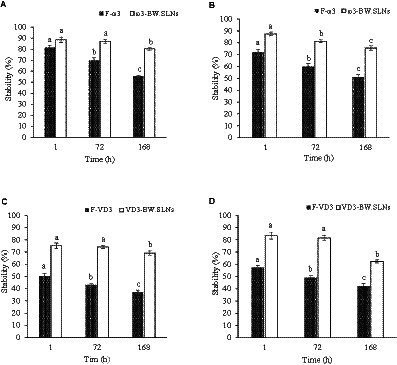
Stability of F- ω3 and entrapped ω3 in BW. SLNs under acidic pH **(A)** and alkaline pH **(B)**. Stability of F-VD3 and entrapped VD3 in BW. SLNs under acidic pH **(C)** and alkaline pH **(D)**.

The stability of free VD_3_ (F-VD_3_), free ω3 (F-ω3), and encapsulated VD_3_ and ω3 was investigated in aqueous solutions with pH 9.0 and 2.0. The stability of F-ω3 at an acidic pH of 2 was found to be 81.5, 69.9 and 55.4% after 1, 72, and 168 h, respectively (Figure 4A). After 168 h of incubation at an acidic pH, entrapped ω3 was more stable and more than 80% of loaded molecules remained intact in nanoparticles in acidic conditions. After 168 h of incubation under alkaline conditions, only 51.3% of the free ω3 were still intact because ω3 was sensitive to an alkaline pH (pH 9) (Figure 4B). During 168 h of incubation at an alkaline pH, BW. SLNs increased the stability of the encapsulated ω3 to 75.5%. In accordance with the present work, Campos et al. (50) demonstrated that using wax or lipids to form a nanoparticle matrix can enhance the stability of entrapped compounds. The susceptibility of free and entrapped VD_3_ to pH changes was higher than that of free and entrapped ω3 molecules. The stability of free VD_3_ molecules was retained at a rate of 50.26% under pH of 2 after 1 h, which subsequently decreased to 37.2% after 168 h of incubation (Figure 4C). Stability of entrapped VD_3_ under acidic conditions was obtained at 75.2, 74.31 and 69.23% during 1, 72 and 168 h, respectively. Similar to acidic pH, alkaline pH had adverse effects on free molecules of VD_3_, and after 168 h, it affected more than 58% of these molecules (Figure 4D). Therefore, it could be inferred that the ω3 and VD_3_ molecules that are trapped within BW. SLNs are shielded from the negative effects of alkaline or acidic pH. Our findings are in agreement with the studies of Qian et al. (51). Their findings demonstrated that beta-carotene degradation is promoted in acidic conditions (pH = 3) (51). Also, in a study conducted by Mitbumrung et al. (52), due to the sensitivity of VD_3_ to acidic pH, the EE of entrapped VD_3_ in Pickering emulsions (10% wt soybean oil-in-water) stabilized by nanofibrillated cellulose reduced at pH 2. In contrast to our findings, Park et al. (53) found encapsulated VD_3_ in nanostructured lipid carriers was stable in acidic conditions, and the EE was about 75% in all pH conditions (pH 2.0, 4.0, and 10), which indicates that pH does not affect it.

### 3.6 Stability of VD_3_/ω3-BW. SLNs under oxidative condition (H_2_O_2_)

**Figure 5:**
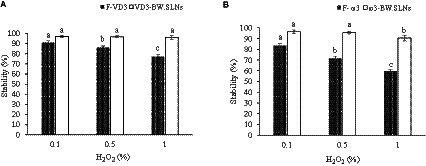
The oxidation stability of F-VD3 and entrapped VD3 **(A)** and F- ω3 and entrapped ω3 **(B)** in BW. SLNs in solutions with different H2O2 levels.

In different concentrations of H_2_O_2_ solutions (0.1, 0.5, and 1% v/v), the oxidation stability of F-VD_3_, F-ω3, and encapsulated VD_3_ and ω3 in BW. SLNs was examined. The results demonstrated that F-VD_3_ and F-ω3 were more sensitive to H_2_O_2_ oxidation (Figures 5A,B). However, VD_3_/ω3-BW. SLNs showed higher oxidation resistance for VD_3_ and ω3, so 96.2 and 90.4% of entrapped VD_3_ and ω3 remained intact in nanoparticles at highly oxidizing conditions (H_2_O_2_: 1% v/v). The incorporation of VD_3_ and ω3 in lipid nucleation, which restricts their exposure to oxidative agents, may be the reason for preventing the oxidation of VD_3_ and ω3 (33). Similar results were obtained in the study of Eratte et al. (54), in which encapsulated ω3 fatty acids in microcapsules of whey protein isolate and gum Arabic showed stronger oxidative stability compared to untreated samples. Also, Xiao et al. (23) used a complex of alginate and ovalbumin to co-encapsulate omega-3 fatty acids with curcumin, lutein, and essential oils, and the highest oxidative stability was obtained in treatments containing omega-3 fatty acids and garlic essential oil during one-month of storage.

### 3.7 Stability of zeta potential and size of VD_3_/ω3-BW. SLNs during the time

**Figure 6:**
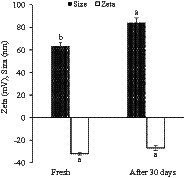
The storage stability of VD3/ω3-BW. SLNs at 30°C for 30 days.

Size and zeta potential of VD_3_/ω3-BW. SLNs suspensions were analyzed after 1 month of storage at 4°C. The findings showed that zeta potential and size were altered from −32 mV and 63.5 nm to −27 mV and 84.2 nm, respectively (Figure 6). However, no aggregation of nanoparticles was observed in the suspensions even after 1 month. This is in agreement with the findings of Andrade Chaves et al. (55), who reported an increase in the size of liposomes loaded with curcumin and vitamin D_3_ during 42 days of storage. In addition, a decrease in zeta potential occurred during the storage periods that, external exposure of phosphate and absorption of OH-from the water environment were mentioned as the reasons for the negative value of zeta potential (55). In a survey conducted on ω3 and α-tocopherol co-encapsulated in a nano lipid carrier, the appropriate zeta potential (−1.1 mV) and particle size (110 nm) were obtained after 75 days of storage at 25°C, which indicated a relatively low tendency of nanoparticles to agglomerate and their good stability (22). Campos et al. (56) stated that mannitol at a concentration of 10% (w/v) was an appropriate cryoprotectant for suspensions of nanoparticles and SLNs containing phenolic compounds during 3 months of storage. In our research, the storage stability of SLNs in suspension was assessed without the use of cryoprotectants, and the nanoparticles remained stable during 30 days of storage.

## 4 Conclusion

In the present study, omega-3 and vitamin D_3_ were successfully co-loaded into beeswax solid lipid nanoparticles. Change in EE of bioactive ingredients depending on the concentration of the core solution was observed. The simultaneous encapsulation of VD_3_ and ω3 at concentrations 5 and 10 mg, respectively, showed the highest encapsulation efficiency and spherical nanocapsules with the lowest size (63.5 nm), which was selected as an optimum formulation. *In vitro*, release study indicated that 19.4 and 9.3% of ω3 and VD_3_ could be absorbed on nanoparticle surfaces and quickly released into the buffer solution. Further, BW. SLNs were effective in protecting both bioactives from oxidative conditions and high pH levels. The VD_3_/ω3-BW. SLNs nanocomplexes have good storage stability and no agglomerate or aggregate was observed after 30 days of storage at 4°C. Hence, nanoparticles showed high stability against harsh conditions, which is important for the use of sensitive nutrients. The co-encapsulated omega-3 and vitamin D_3_ in beeswax solid lipid nanoparticles could efficiently be used in the food industry to develop functional products. The increasing demand for functional foods containing health-promoting ingredients strengthens the importance of research in this field. There are still some limits to our knowledge about the use of other waxes in different proportions, with beeswax as carriers to encapsulate bioactive molecules. Since beeswax nanoparticles are a good carrier and have a suitable loading capacity, it is suggested to load various fat-soluble vitamins, essential oils, antibiotic compounds, and post-biotic compounds into them in future research.

## Data availability statement

The original contributions presented in the study are included in the article/supplementary material, further inquiries can be directed to the corresponding authors.

## Author contributions

MS: Conceptualization, Data curation, Investigation, Validation, Writing – original draft. RG: Conceptualization, Data curation, Visualization, Writing – original draft. SS: Methodology, Project administration, Supervision, Writing – review & editing. EK: Formal analysis, Methodology, Resources, Validation, Writing – review & editing. NM-M: Conceptualization, Validation, Visualization, Writing – review & editing.
